# Sex differences in intra‐articular treatment outcomes for knee osteoarthritis: Current evidence and research gaps: A systematic review and meta‐analysis

**DOI:** 10.1002/jeo2.70432

**Published:** 2025-09-23

**Authors:** Gae Fattini Fellini, Giacomo A. Fumagalli, Andrea Piano, Alessandro Bensa, Giuseppe Filardo

**Affiliations:** ^1^ Service of Orthopaedics and Traumatology, Department of Surgery EOC Lugano Switzerland; ^2^ Università della Svizzera Italiana Faculty of Biomedical Sciences Lugano Switzerland

**Keywords:** gender, intra‐articular, knee, osteoarthritis, sex

## Abstract

**Purpose:**

The aim of this systematic review and meta‐analysis was to quantify and compare the evidence on sex‐specific outcomes following intra‐articular injections of corticosteroids (CS), hyaluronic acid (HA), platelet‐rich plasma (PRP) and cell‐based therapies in patients affected by knee osteoarthritis (OA).

**Methods:**

A literature search was conducted on PubMed, Cochrane and Web of Science in October 2024 according to PRISMA guidelines. Inclusion criteria were clinical studies of any level of evidence, a minimum of six patients, English language, no time limitations, on the use of intra‐articular CS, HA, PRP and cell‐based therapies for knee OA treatment. A meta‐analysis was conducted for each product on the outcomes with sufficient data at short‐term (<3 months) and mid/long‐term (≥3 months) follow‐ups.

**Results:**

A total of 848 studies were included reporting on 99,443 patients (61.5 ± 24.7 years old, 65.8% women, 34.2% men). Out of these, only 21 articles (2.5% of the total, 2265 patients) presented sex‐disaggregated data, including four studies with a CS treatment group (1.9% of CS patients), three with a HA group (0.2% of HA patients), 11 with a PRP group (5.9% of PRP patients) and eight with a cell‐based therapy group (10.2% of cell‐based therapies patients). The meta‐analysis was performed on 15 studies (1387 patients) and did not show any statistically significant difference of VAS and WOMAC improvements at short‐ and mid/long‐term follow‐ups for any of the four injectable treatment approaches analysed.

**Conclusion:**

There is a staggering lack of sex‐disaggregated data in studies evaluating the most used intra‐articular injective therapies for knee OA, with only 2.5% analysing women‐ and men‐specific outcomes for CS, HA PRP and cell‐based therapies. The analysis of this limited amount of sex‐disaggregated data did not show statistically significant differences between women and men for any of the investigated injectable approaches. The substantial lack of disaggregation hinders the understanding of potential sex‐specific effects of these treatments and underscores the need for a shift in data reporting in this field, with more robustly designed studies incorporating sex‐stratified analyses.

**Level of Evidence:**

Level I.

**Abbreviations**
CI: confidence interval
CS: corticosteroids
HA: hyaluronic acid
KL: Kellgren–Lawrence
MD: mean difference
NIH: National Institutes of Health
OA: osteoarthritis
PRISMA: preferred reporting items for systematic reviews and meta‐analysis
PROMs: patient‐reported outcome measures
PRP: platelet‐rich plasma
TKA: total knee arthroplasty
VAS: visual analogue scale
WOMAC: Western Ontario and McMaster Universities Osteoarthritis Index

## INTRODUCTION

Knee osteoarthritis (OA) is a multifactorial disease causing significant impairment to the affected patients, with a significant burden on healthcare systems worldwide [3, 31]. When conservative treatments fail, intra‐articular injections offer a minimally invasive alternative for pain management and functional improvement. Among the available options, corticosteroids (CS) and hyaluronic acid (HA) are the most commonly used, with platelet‐rich plasma (PRP) and cell‐based therapies becoming increasingly popular [10, 11, 12, 14, 27, 44]. Despite the growing interest on the clinical effectiveness of these injective strategies for knee OA, with a considerable amount of published studies investigating this aspect, the variability in the obtained results underpins the need to investigate patient‐specific aspects influencing the treatment response [4, 13, 15, 44, 59].

The clinical results of the most used intra‐articular injective products for knee OA may depend on the interplay of several factors, ranging from OA severity to patient characteristics, although their role in this chronic pathology has not been well defined, yet [17, 42]. Among these, the scarce evidence on sex‐related differences fuels an ongoing debate on the impact on safety and effectiveness of intra‐articular injective treatments for knee OA. Sex differences are well‐documented across a wide spectrum of diseases, and recognising the influence of sex on disease prevalence, presentation and treatment response is paramount for optimising patient care and advancing precision medicine [8, 58]. This is particularly relevant in the context knee OA, where these differences may significantly affect disease burden and treatment outcomes [45]. In fact, knee OA exhibits a higher prevalence in women, who tend to experience more severe symptoms, greater utilisation of NSAIDs, and less favourable outcomes with certain therapeutic interventions, including invasive approaches like total knee arthroplasty (TKA) [20, 56]. Despite the growing awareness on this topic, research outcomes often lack disaggregation by sex, hindering sex‐specific analyses and thus the development and implementation of personalised treatment strategies [7]. In this light, a systematic review and meta‐analysis comparing the outcomes of women and men would be of clinical relevance, offering valuable information on the possible sex‐related differences in knee OA to help optimising the management of this condition in the clinical practice.

The aim of this systematic review and meta‐analysis was to quantify and compare the evidence on sex‐specific outcomes following intra‐articular injections of CS, HA, PRP and cell‐based therapies in patients affected by knee OA, with the hypothesis that the sex‐specific data provided in the available literature will help elucidating this important but often overlooked aspect for the clinical practice.

## MATERIALS AND METHODS

### Literature search

**Figure 1:**
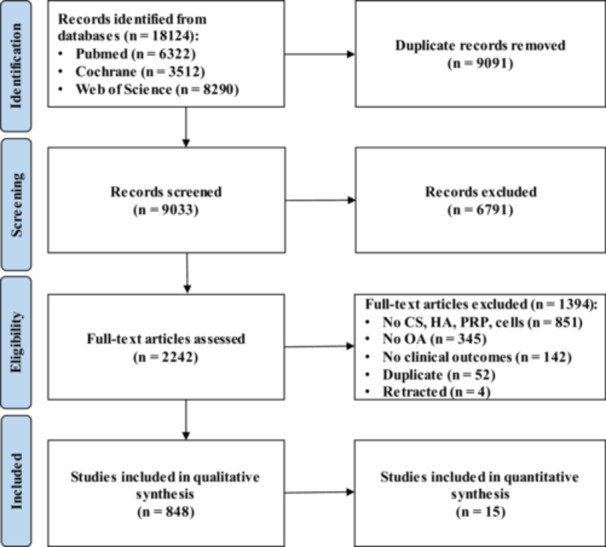
PRISMA flow diagram.

A systematic review of the literature was performed on the intra‐articular injective use of CS, HA, PRP and cell‐based therapies for the treatment of knee OA [50, 51]. The literature search was conducted on the PubMed, Cochrane and Web of Science databases without any filter on 10 October 2024, using the following search string: (inject* OR intra‐articular* OR infiltrat*) AND (osteoarthritis OR OA) AND (knee). The trial was registered on PROSPERO (ID: CRD42025642857) and the guidelines for preferred reporting items for systematic reviews and meta‐analysis (PRISMA) were used (Figure 1) [40].

### Studies selection and data extraction

The screening and analysis processes were conducted separately by two independent observers (A.P. and G.A.F.) and any discrepancies between them were resolved by discussion and consensus with a third author (A.B.). First, the articles were screened by title and abstract. The inclusion criteria were clinical studies of any level of evidence involving a minimum of six patients, written in English language, with no time limitations, on the use of intra‐articular injections of CS, HA, PRP and cell‐based therapies for knee OA treatment. The exclusion criteria were studies published in languages other than English, literature reviews, in vitro articles, preclinical in vivo studies, case reports, case series with less than six patients, congress abstracts, studies not reporting clinical outcomes, studies reporting the combined use of CS, HA, PRP or cell‐based therapies without analysing the effect of the individual products, clinical studies on subchondral injections or on joints different from the knee, or other diseases different from OA. The initial screening was performed manually based on the title and abstract. Subsequently, the full texts of the selected articles were screened, with further exclusions according to the previously described criteria. Relevant data were extracted from the included studies full texts, figures and tables. The extracted data included authors' name, publication year, journal, study design, patient demographics (sex, age), injected product, sample size, patient‐reported outcome measures (PROMs) and adverse events. These data were then summarised and analysed to address the study objectives. A meta‐analysis was conducted on the PROMs at two distinct follow‐ups: short‐term (<3 months) and mid/long‐term (≥3 months). Each outcome was included in the meta‐analysis only if it was reported by at least two studies for a specific follow‐up.

### Risk of bias and quality of evidence

The risk of bias of each included article was assessed using the standardised critical appraisal tools developed by the Joanna Briggs Institute (JBI) [41]. These tools are tailored to specific study designs (e.g., randomised controlled trials, cohort studies, case series etc.) and provide a structured approach to evaluate aspects such as study validity, reliability and relevance. The overall quality of evidence for each outcome was graded according to the Grading of Recommendations Assessment, Development and Evaluation (GRADE) guidelines for high, moderate, low and very low levels of evidence [53]. The risk of bias and the quality of evidence evaluations were performed separately by two independent observers (A.P. and G.A.F.) and any discrepancies between them were resolved by discussion and consensus with a third author (A.B.).

### Statistical analysis

The statistical analysis and Forest plotting were performed according to Neyeloff et al. using the Meta XL tool for Microsoft Excel [44]. The analysis was performed using random effects (DerSimonian & Laird) for the weighted mean differences (MD). The *I* ^2^ statistic for heterogeneity was used as well as the *Q* statistic. The weighted MD (delta) was used to calculate the *Z*‐test statistic for continuous outcomes. The 95% confidence intervals (95% CIs) of the delta were then derived and if the 95% CI excluded zero, then the meta‐analysis of interest had a statistically significant result at 0.05 level of significance. In addition, the derived results were used to define the test statistic *Z* = delta/standard error which is *N* (0, 1), and therefore its corresponding *p*‐value was used to confirm or negate the result of the same meta‐analysis.

## RESULTS

### Characteristics of the included studies and patients

**Figure 2:**
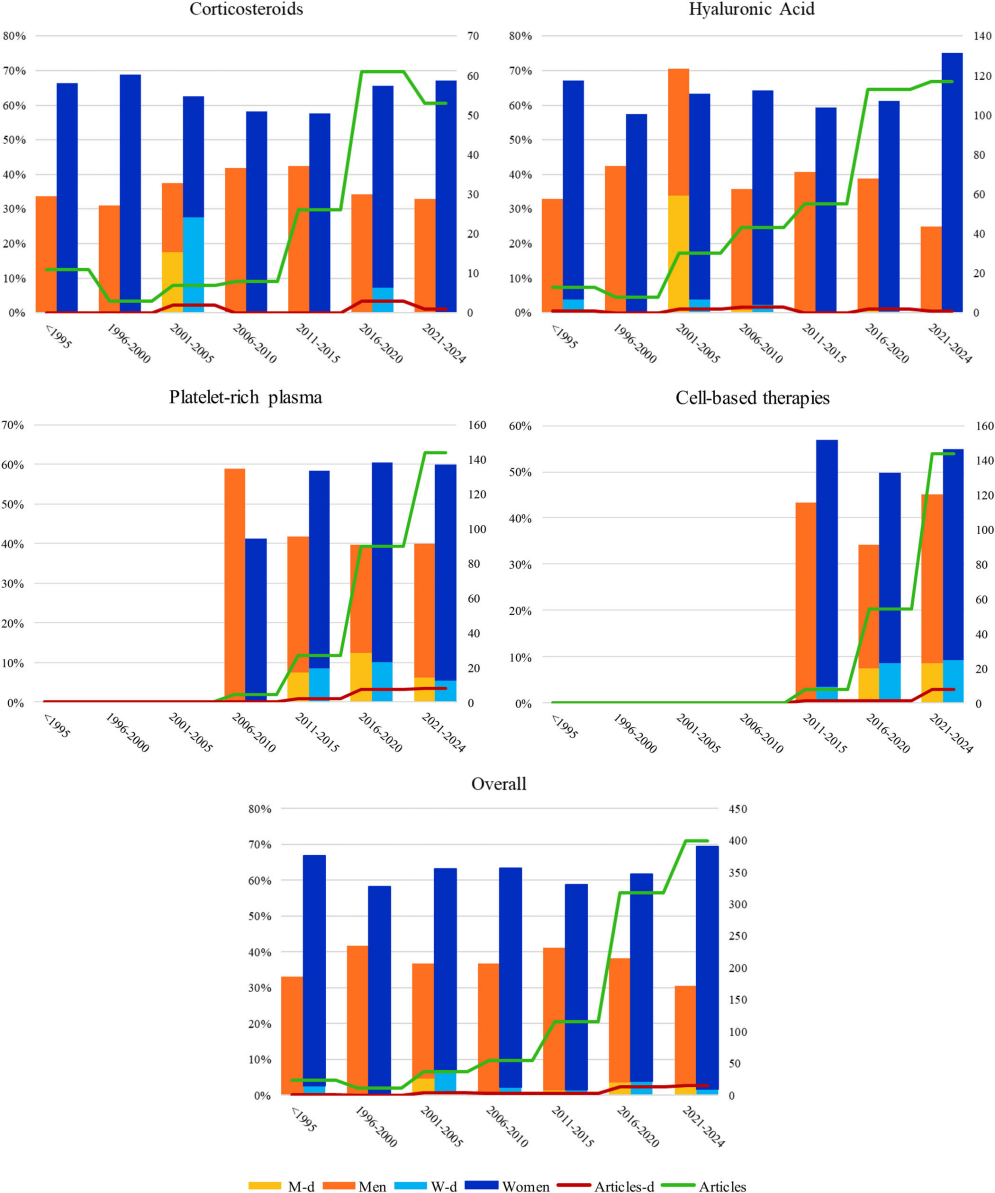
The red line indicates the number of articles providing sex‐specific data for intra‐articular injections in knee osteoarthritis. The yellow bars show the percentage of men with specific data (M‐d) relative to the total number of men, while the light blue bars indicate the percentage of women with specific data (W‐d) relative to the total number of women. Data are categorised and presented in 5‐year intervals across different treatment types: corticosteroids, platelet‐rich plasma, hyaluronic acid and cell‐based therapies, as well as overall trends.

A total of 18,125 articles was retrieved. After removing duplicates, 9033 articles were available for screening. The screening of titles and abstracts led to the identification of 2242 articles for full‐text screening. After eliminating the articles according to the inclusion and exclusion criteria applied to the full texts screened, 848 studies, published between 1959 and 2024, were included (Figure 1). The data extracted from the relative papers were included in the qualitative and quantitative data syntheses. A total of 99,443 patients (mean age 61.5 ± 24.7 years) were enrolled in the 848 studies analysed. In 76 articles (9.0%), for a total of 4584 patients (4.6% of the total), sex was not reported, while for 772 articles (91.0%), for a total of 94,910 patients (95.4% of the total, 65.8% women, 34.2% men), sex was defined. Out of these, 12 studies included only women (1.4%) and two studies (0.2%) included only men. Among the 848 full‐text articles, 21 articles (2.5%), for a total of 2265 patients (2.3% of total, 54.0% women, 46.0% men) presented sex‐disaggregated data and, among these, 20 articles (2.4%), for a total of 2185 patients (2.2% of total, 54.0% women, 46.0% men) provided sex‐disaggregated data referring to a common clinical outcome. The percentage of articles providing sex‐specific data for intra‐articular injections in knee OA remains negligible despite a minor increase observed starting from 2016 (Figure 2).

#### CS

In 169 articles, a total of 12,185 patients (mean age 61.8 ± 6.8 years) received CS injections. In 23 articles (13.6%), sex was not reported, while for 146 articles (86.4%), sex was defined. Out of these, two studies included only women. Among the 169 articles, four articles (2.4%), for a total of 237 patients (1.9% of total CS patients, 74.6% women, 25.4% men) provided sex‐disaggregated data and, among these, three articles (1.8%), for a total of 197 patients (1.6% of total CS patients, 74.6 women, 25.4% men) provided disaggregated data by sex referring to a clinical outcome.

#### HA

In 379 articles, a total of 60,708 patients (mean age 63.9 ± 35.1 years) received HA injections. In 37 articles (9.8%), sex was not reported, while for 342 articles (90.2%) sex was defined. Out of these, five studies (1.3%) included only women and one study (0.3%) included only men. Among the 379 articles, three articles (0.8%), for a total of 122 patients (0.2% of total HA patients, 56.6% women, 43.4% men) presented sex‐disaggregated data and, among these, two articles (0.5%), for a total of 82 patients (0.1% of total HA patients, 52.4% women, 47.6% men) articles provided disaggregated data by sex referring to a clinical outcome.

#### PRP

In 265 articles, a total of 18,547 patients (mean age 57.7 ± 6.2 years) received PRP injections. In 19 articles (7.2%), sex was not stated, while for 246 articles (92.8%) sex was defined. Out of these, six studies (2.3%) included only women. Among the 265 articles, 11 articles (4.2%), for a total of 1093 patients (5.9% of total PRP patients, 50.7% women, 49.3% men), provided disaggregated data by sex referring to a clinical outcome.

#### Cell‐based therapies

In 148 articles, a total of 8003 patients (mean age 59.0 ± 6.4 years) received cell‐based therapies injections. In 15 articles (10.1%), sex was not stated, while for 133 articles (89.9%) sex was defined. Out of these, two studies included only women (1.4%). Among the 148 articles, six articles (4.1%), for a total of 813 patients (10.2% of total cell‐based therapies patients, 53,2% women, 46.8%) articles provided disaggregated data by sex referring to a clinical outcome.

### Meta‐analysis

**Figure 3:**
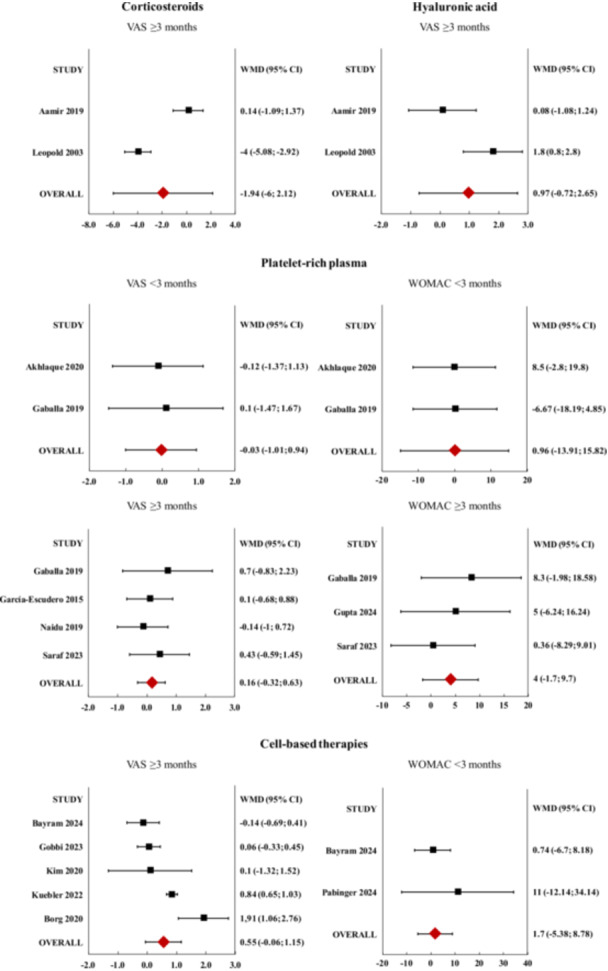
Forest plots of the individual studies and weighted mean difference (WMD) of the visual analogue scale (VAS) for pain improvement and Western Ontario and McMaster Universities Osteoarthritis index (WOMAC) improvement at short‐term follow‐up (<3 months) and mid/long‐term follow‐up (≥3 months) for corticosteroids, hyaluronic acid, platelet‐rich plasma, and cell‐based‐therapies. No statistically significant differences of VAS and WOMAC improvements were detected for any of the four treatment approaches at any follow‐up.

A meta‐analysis was performed on 15 studies, encompassing 1387 patients (55.4% women, 44.6% men). The PROMs with sufficient data for the meta‐analysis included the visual analogue scale (VAS) for pain and the Western Ontario and McMaster Universities Osteoarthritis index (WOMAC). The minimal clinically important differences (MCID) reported in the literature for each score are 6.4/96 for the WOMAC score and 1.4/10 for the VAS pain score [6, 54]. The characteristics of the studies included in the meta‐analysis are reported in Table 1 and the results are represented in Figure 3.

**Table 1:** Patient and treatment characteristics of the studies included in the meta‐analysis.

Study	Year	Journal	Treatment	Patients	Women	Men	Age	Outcomes
Aamir et al. [2]	2019	*Med Forum Month*	CS	40	40	40	59.6 ± 7.6	VAS
			HA	40			59.4 ± 8.3	
Akhlaque et al. [5]	2020	*J Pak Med Assoc*	PRP	50	26	24	59.6 ± 9.6	VAS
Bayram et al. [9]	2024	*Agri*	Autologous adipose tissue	165	103	62	61.3 ± 11.4	VAS, WOMAC
Borg et al. [19]	2020	*Stem Cells Int*	Microfragmented adipose tissue	386	192	194	65.5 ± 12.0	VAS
Gaballa et al. [23]	2019	*Egyptian Rheumatol*	PRP	20	15	5	53.6 ± 4.6	VAS, WOMAC
García‐Escudero et al. [24]	2015	*Plos One*	Autologous conditioned serum	118	75	43	59.1	VAS
Gobbi et al. [26]	2023	*Eur J Orthop Surg Traumatol*	Microfragmented adipose tissue	40	23	17	62.8 ± 13.0	VAS
Gupta et al. [28]	2024	*Cureus*	PRP	40	25	15	NR	WOMAC
Kim et al. [34]	2020	*Appl Sci*	Bone marrow aspirate concentrate	25	16	9	67.5	VAS
Kuebler et al. [36]	2022	*Stem Cells Int*	Bone marrow aspirate concentrate	160	83	77	63.2 ± 1.0	VAS
Leopold et al. [38]	2003	*J Bone Joint Surg Am*	CS	50	28	22	66.0 ± 11.6	VAS, WOMAC
			HA	50	28	22	64.0 ± 12.4	
Naidu et al. [43]	2019	*J Evolution Med Dent Sci*	PRP	50	32	18	52.7	VAS
Pabinger et al. [46]	2024	*Sci Rep*	Bone marrow aspirate concentrate	29	11	18	58.0 ± 18	WOMAC
Saraf et al. [55]	2023	*J Clin Orthop Trauma*	PRP	65	37	28	54.4 ± 8.7	VAS, WOMAC
Sibillin et al. [57]	2022	*ANZ J Surg*	PRP	59	35	24	61.2 ± 12.6	WOMAC

Abbreviations: CS, corticosteroids; HA, hyaluronic acid; NR, not reported; PRP, platelet‐rich plasma; VAS, visual analogue scale for pain; WOMAC: Western Ontario and McMaster Universities Arthritis Index.

#### CS

*VAS mid/long‐term*: The analysis of mid/long‐term VAS outcomes, based on two studies including 90 patients (56.0% women, 44.0% men), did not yield statistically significant differences in the treatment results [2, 38].

#### HA

*VAS mid/long‐term*: The analysis of long‐term VAS outcomes, based on two studies including 90 patients (56.0% women, 44.0% men), did not yield statistically significant differences in the treatment results [2, 38].

#### PRP

*VAS short‐term*: The analysis of short‐term VAS outcomes, based on two studies including 70 patients (58.6% women, 41.4% men), did not yield statistically significant differences in the treatment results [5, 23].

*VAS mid/long‐term*: The analysis of long‐term VAS outcomes, based on four studies including 253 patients (62.8% women, 37.1% men), did not yield statistically significant differences in the treatment results [23, 24, 43, 55].

*WOMAC short‐term*: The analysis of short‐term WOMAC outcomes, based on two studies including 79 patients (63.3% women, 36.7% men), did not yield statistically significant differences in the treatment results [23, 57].

*WOMAC mid/long‐term*: The analysis of long‐term WOMAC outcomes, based on three studies including 125 patients (61.6% women, 38.4% men), did not yield statistically significant differences in the treatment results [23, 28, 55].

#### Cell‐based therapies

*VAS mid/long‐term*: The analysis of long‐term VAS outcomes, based on five studies including 776 patients (53.7% women, 46.3% men), did not yield statistically significant differences in the treatment results [9, 19, 26, 34, 36].

*WOMAC short‐term*: The analysis of short‐term WOMAC outcomes, based on two studies including 202 patients (58.8% women, 41.2% men), did not yield statistically significant differences in the treatment results [9, 46].

### Risk of bias and quality of evidence

The evaluation using the JBI Critical Appraisal Tools showed an overall moderate risk of bias across the included studies. A summary of the risk of bias assessment is provided in Table 2. The GRADE showed that the level of evidence of the results was very low in almost all the outcomes, with only two outcomes having a low level of evidence. A summary of the quality of evidence assessment of the meta‐analysis outcomes is provided in Table 3.

**Table 2:** Joanna Briggs Institute critical appraisal tools evaluation.

Article	Study type	D1	D2	D3	D4	D5	D6	D7	D8	D9	D10	D11	D12	D13
Aamir 2019	Randomised controlled trial (RCT)	Unclear	Unclear	Yes	No	No	Yes	Unclear	Yes	Unclear	Yes	Yes	Yes	Yes
Gaballa 2019	RCT	Unclear	Unclear	Yes	No	No	Yes	Unclear	Yes	Yes	Yes	Yes	Yes	Yes
Gobbi 2023	RCT	Yes	No	Yes	No	No	Yes	Yes	Yes	Yes	Yes	Yes	Yes	Yes
Leopold 2003	RCT	Yes	Yes	Yes	No	No	Yes	Yes	Yes	Yes	Yes	No	Yes	Yes
Borg 2021	Cohort	Yes	N/A	Yes	Yes	Yes	Yes	Yes	Yes	Yes	Yes	Yes		
García‐Escudero 2015	Cohort	No	N/A	Yes	Yes	Yes	Yes	Yes	Yes	Yes	Yes	Yes		
Kuebler 2022	Cohort	No	Yes	Yes	Yes	No	Yes	Yes	Yes	Yes	Yes	Yes		
Pabinger C 2024	Cohort	No	N/A	Yes	Yes	Yes	Yes	Yes	Yes	Yes	Yes	Yes		
Sibillin 2022	Cohort	No	N/A	Yes	Yes	Yes	Yes	Yes	Yes	Yes	Yes	Yes		
Bayram 2024	Case‐series	Yes	Yes	Yes	Yes	Yes	Yes	Yes	Yes	Yes	Yes			
Gupta 2024	Case‐series	Yes	Yes	Yes	Yes	Yes	Yes	Yes	Yes	Yes	Yes			
Kim GB 2020	Case‐series	Yes	Yes	Yes	Yes	Yes	Yes	Yes	Yes	Yes	Yes			
Naidu 2019	Case‐series	Yes	Yes	Yes	No	Yes	Yes	Yes	Yes	Yes	Yes			
Saraf 2023	Case‐series	Yes	Yes	Yes	Yes	Yes	Yes	Yes	Yes	Yes	Yes			
Akhlaque 2020	Quasi‐experimental	Yes	No	N/A	Yes	Yes	Yes	Yes	Yes	Yes				

**Table 3:** Grading of recommendations assessment, development and evaluation.

Outcomes	Risk of bias	Inconsistency	Indirectness	Imprecision	Publication bias	Other	Quality of evidence
VAS CSmid/long‐term	No	Serious	Serious	Serious	No	No	Very low
VAS HA mid/long‐term	No	Serious	Serious	Serious	No	No	Very low
VAS PRP short‐term	Serious	Serious	Serious	Serious	No	No	Very low
VAS PRP mid/long‐term	Serious	Serious	No	No	No	No	Low
WOMAC PRP Short‐term	Serious	Serious	Serious	Serious	No	No	Very low
WOMAC PRP mid/long‐term	Serious	Serious	Serious	Serious	No	No	Very low
VAS cells mid/long‐term	Serious	Serious	No	No	No	No	Low
WOMAC cells Short‐term	Serious	Serious	Serious	Serious	No	No	Very low

Abbreviations: VAS, visual analog scale for pain; WOMAC, Western Ontario and McMaster Universities Osteoarthritis Index.

## DISCUSSION

The main finding of this systematic review and meta‐analysis is the staggering lack of sex‐disaggregated data in studies evaluating the most used intra‐articular injective therapies for knee OA, with only 2.5% of the studies analysed providing women‐ and men‐specific outcomes data for CS, HA, PRP and cell‐based therapies. The analysis of this limited amount of sex‐disaggregated data did not show statistically significant differences between women and men for any of the investigated injectable approaches. The lack of disaggregation hinders the understanding of potential sex‐specific effects of these treatments and underscores the need for a shift in data reporting in this field, with more robustly designed studies incorporating sex‐stratified analyses.

Sex differences are well‐documented across orthopaedic conditions, with a relevant impact on epidemiology, presentation and therapeutic implications [8, 58]. According to Peshkova et al., the well‐known higher prevalence of knee OA in women is multifactorial, involving anatomical, biomechanical, hormonal and molecular aspects [49]. These differences underpin the importance of systematically investigating the sex‐related aspects of this disease. Despite the progress in sex‐specific analyses, most orthopaedic studies still do not perform data disaggregation. Previous reviews have emphasised general failures in sex reporting in OA and orthopaedics but did not provide quantitative or treatment‐specific insights. Espinosa‑Salas et al. found only one trial reporting sex‐specific outcomes in nine systemic OA drug studies, concluding that significance of any sex differences remains unclear due to data paucity [21]. Hettrich et al. reported an increase in sex‐specific analyses in leading high‐impact orthopaedic journals, from 19% to 30% between 2000 and 2010 [29]. Similarly, Gianakos et al. found that only 34% of studies published in 2016 in the same five journals, plus one additional journal, included sex as a variable in multifactorial statistical models, with 39% reporting differences in outcomes between male and female patients [25].

These sex‐based differences pertain various orthopaedic treatments. Women exhibit distinct patterns in treatment utilisation and response, often experiencing lower satisfaction and higher reintervention rates following cartilage surgery [22]. Women undergoing TKA tend to seek treatment at a more advanced disease stage and with greater functional disability. Furthermore, disparities persist postoperatively, affecting functional outcomes [47]. A prospective cohort study demonstrated that women who undergo high tibial osteotomy transition to TKA sooner than men [32]. Despite the growing evidence corroborating the relevance of considering sex‐related differences in the the outcomes of orthopaedic conditions, the current scientific literature presents a significant lack of studies specifically investigating this aspect in intra‐articular injection therapies for knee OA, as documented by the present study.

This systematic review and meta‐analysis aimed at investigating this gap by evaluating the effectiveness of the most used intra‐articular treatments, namely CS, HA, PRP and cell‐based therapies, through the analysis of VAS pain and WOMAC score improvements at short‐ and mid/long‐term follow‐ups. The results did not show any statistically significant differences between men and women across all treatment modalities for both VAS and WOMAC at any follow‐up. However, this should not be interpreted as evidence of equivalence. In fact, the number of studies reporting the disaggregated data necessary to perform the meta‐analysis was considerably limited and several of the effect estimates were associated with wide confidence intervals, indicating that potentially meaningful differences may not reach statistical significance. Overall, these findings suggest that the current evidence base may be underpowered to detect clinically relevant sex differences. As such, it is possible that some of the outcomes did not reach statistical significance in the event of a true positive effect. This considerably restricts the strength of the conclusions supported by these results, warranting caution in their interpretation and underlining another major finding in this field about the need to address the lack of sex‐disaggregated data.

These results demonstrated that the lack of sex‐disaggregated data in orthopaedics is particularly evident in intra‐articular injective therapies for knee OA. In fact, the most striking finding of the present study is that only 2.5% of the studies investigating the most used injective treatments for knee OA disaggregated data according to the patients' sex, representing less than 2.3% of the total number of patients studied. This impressive lack of disaggregation regards all the products studied, but with some differences. CS and HA were the options presenting the lowest number of sex‐disaggregated studies, 1.9% and 0.2% respectively. This may be related to the fact that these are the oldest products used in the clinical practice and therefore the initial studies investigating these treatments were performed in a time period where the awareness of the importance of sex‐disaggregated analyses was more limited than nowadays. However, the results of this meta‐analysis showed that even in recent years there has been no significant increase in the number of studies reporting sex‐disaggregated data for these two products.

Orthobiologic treatments have gained attention more recently, particularly when conventional intra‐articular therapies such as CS and HA fail to provide satisfactory results. These therapies leverage the regenerative and immunomodulatory potential of platelets and mesenchymal cells, aiming not only at alleviating symptoms but also at modifying the natural OA progression, as supported by preclinical studies demonstrating disease‐modifying effects of both blood‐derived and cell‐based products [17, 18, 35, 37, 48]. Compared to CS and HA, PRP and cell‐based therapies have been the subject of a relatively higher number of studies incorporating sex‐disaggregated analyses, and in recent years a modest yet encouraging trend has emerged towards an increase in the stratification of treatment effects based on sex. Despite this progress, the overall proportion of patients with disaggregated data remains considerably low, 5.9% of patients in PRP studies and 10.2% in cell‐based therapy studies, highlighting the urgent need for further research to close this gap and implement personalised treatment strategies for knee OA management.

Previous meta‐analyses examined the efficacy and safety of CS, HA, PRP and cell therapies without sex‐based analysis [16, 33, 39, 52]. While these studies remain informative, none systematically explored sex differences or investigated on how sex‐specific reporting has evolved across therapies. The present study fills this gap by quantifying, comparing and meta‐analysing sex‐specific outcomes across injective treatments. The lack of sex‐specific data resulted particularly severe in the field of injective treatments for knee OA, but this problem concerns also other areas of the orthopaedic research and of the medical research in general. In 2015, the National Institutes of Health (NIH) issued a notice emphasising the need to consider sex as a biological variable in both animal and human studies [1]. To this aim, a further distinction should be considered to better embrace the complexity and relevance of this study area. Women and men are characterised by both sex and gender. Sex is a biological variable, while gender refers to social, cultural and psychological traits linked to human males and females. Sex, gender and their interactions can influence health and disease outcomes [30]. As such, a more refined and detailed approach to sex‐ and gender‐based research is necessary to enhance our understanding of their role in knee OA management.

This systematic review meta‐analysis screened all the available evidence on the most used injectable treatments for knee OA by analysing 848 studies on almost 100,000 patients. Still, this study presents some limitations that require consideration. First, the limited availability of disaggregated data impacts the overall quality of evidence and may explain the lack of statistically significant sex‐related differences in the investigated treatment outcomes. This remarkable lack of disaggregated data prevents from drawing solid conclusions on the possible sex‐related differences in the outcomes of the most used injectable treatments for knee OA patients, underscoring the need for future studies addressing this aspect very often overlooked in the available literature. Additionally, the heterogeneity in study designs, treatment protocols and outcome measures limits comparability across studies. The lack of systematic data disaggregation also prevented a deeper exploration of sex‐related differences in treatment response, such as complication rates. Furthermore, a binary representation of sex and gender can lead to the exclusion of transgender, genderfluid and intersex individuals, and a simple disaggregation by sex may obscure other less evident yet important aspects, such as socioeconomic status and ethnic background, failing to adequately address the needs of different groups of women and men. Finally, funnel plot analysis was not performed due to the small number of studies per outcome as per Cochrane guidelines, limiting the possibility to assess the publication bias. Given that only 2.5% of studies reported sex‐disaggregated outcomes, there is likely a strong publication bias against such analyses, further emphasising the need for systematic improvement in reporting practices. Despite these limitations, the present systematic review and meta‐analysis was able to provide important results, identifying a staggering lack of sex‐disaggregated data in studies evaluating the most used intra‐articular injective therapies for knee OA, which represents a crucial aspect to consider for the design of further studies and future research efforts aimed at improving the management of this condition in clinical practice. Moreover, this study adds the quantification of sex‐disaggregated data with a meta‐analysis on the most commonly used injective therapies for knee OA, which was lacking in previous literature reports, therefore adding an important element to the discussion on this topic. Future studies should prioritise the integration of sex‐specific analyses to improve the personalisation of intra‐articular treatments for knee OA, ensuring that therapeutic strategies are optimised for men, women and gender‐diverse people. Longitudinal studies with larger cohorts and standardised methodologies are necessary to clarify potential differences in efficacy and safety and guide clinical decision‐making, with potential implications for regulatory bodies, clinical trial design and orthopaedic practice guidelines. Addressing these gaps will be crucial to advance precision medicine in orthopaedics and enhance patient outcomes in knee OA management.

## CONCLUSION

There is a staggering lack of sex‐disaggregated data in studies evaluating the most used intra‐articular injective therapies for knee OA, with only 2.5% analysing women‐ and men‐specific outcomes for CS, HA, PRP and cell‐based therapies. The analysis of this limited amount of sex‐disaggregated data did not show statistically significant differences between women and men for any of the investigated injectable approaches. The substantial lack of disaggregation hinders the understanding of potential sex‐specific effects of these treatments and underscores the need for a shift in data reporting in this field, with more robustly designed studies incorporating sex‐stratified analyses.

## AUTHOR CONTRIBUTIONS

**Gae Fattini Fellini**: Data curation; formal analysis; investigation; visualisation; writing—original draft. **Giacomo A. Fumagalli**: Data curation; formal analysis; investigation. **Andrea Piano**: Data curation; formal analysis; investigation. **Alessandro Bensa**: Methodology; investigation; supervision; writing—review and editing. **Giuseppe Filardo**: Conceptualisation; project administration; supervision; writing—review and editing.

## CONFLICT OF INTEREST STATEMENT

The authors declare no conflict of interest.

## ETHICS STATEMENT

Not applicable.

## Data Availability

No additional data were generated for this review. The data are found in the referenced papers. Data collection forms are available upon request.
